# Genomic miscellany and allelic frequencies of *Plasmodium falciparum msp-1*, *msp-2* and *glurp *in parasite isolates

**DOI:** 10.1371/journal.pone.0264654

**Published:** 2022-03-08

**Authors:** Ibrar Ullah, Asifullah Khan, Muhammad Israr, Mohibullah Shah, Sulaiman Shams, Waliullah Khan, Muzafar Shah, Muhammad Siraj, Kehkashan Akbar, Tahira Naz, Sahib Gul Afridi

**Affiliations:** 1 Department of Biochemistry, Abdul Wali Khan University Mardan, Mardan, Pakistan; 2 Department of Forensic Sciences, University of Swat, Swat, Pakistan; 3 Department of Biochemistry, Bahauddin Zakariya University Multan, Multan, Pakistan; 4 Department of Chemistry, Abdul Wali Khan University Mardan, Mardan, Pakistan; 5 Centre for Animal Sciences & Fisheries, University of Swat, Swat, Pakistan; 6 Department of Zoology, Abbottabad University of Science and Technology, Abbottabad, Pakistan; 7 Department of Biochemistry, Abbottabad International Medical College, Abbottabad, Pakistan; Instituto Rene Rachou, BRAZIL

## Abstract

**Introduction:**

The genomic miscellany of malaria parasites can help inform the intensity of malaria transmission and identify potential deficiencies in malaria control programs. This study was aimed at investigating the genomic miscellany, allele frequencies, and MOI of *P*. *falciparum* infection.

**Methods:**

A total of 85 *P*. *falciparum* confirmed isolates out of 100 were included in this study that were collected from *P*. *falciparum* patients aged 4 months to 60 years in nine districts of Khyber Pakhtunkhwa Province. Parasite DNA was extracted from 200µL whole blood samples using the Qiagen DNA extraction kit following the manufacturer’s instructions. The polymorphic regions of *msp-1*, *msp-2* and *glurp* loci were genotyped using nested PCR followed by gel electrophoresis for amplified fragments identification and subsequent data analysis.

**Results:**

Out of 85 *P*. *falciparum* infections detected, 30 were *msp-1* and 32 were *msp-2* alleles specific. Successful amplification occurred in 88.23% (75/85) isolates for *msp-1*, 78.9% (67/85) for *msp-2* and 70% (60/85) for *glurp* gene. In *msp-1*, the K1 allelic family was predominantly prevalent as 66.66% (50/75), followed by RO33 and MAD20. The frequency of samples with single infection having only K1, MAD20 and RO33 were 21.34% (16/75), 8% (6/75), and 10.67% (8/75), respectively. In *msp-2*, both the FC27 and 3D7 allelic families revealed almost the same frequencies as 70.14% (47/67) and 67.16% (45/67), respectively. Nine *glurp* RII region alleles were identified in 60 isolates. The overall mean multiplicity of infection for *msp* genes was 1.6 with 1.8 for *msp-1* and 1.4 for *msp-2*, while for *glurp* the MOI was 1.03. There was no significant association between multiplicity of infection and age groups (Spearman’s rank coefficient = 0.050; *P* = 0.6) while MOI and parasite density correlated for only *msp-2* allelic marker.

**Conclusions:**

The study showed high genetic diversity and allelic frequency with multiple clones of *msp-1*, *msp-2* and *glurp* in *P*. *falciparum* isolates in Khyber Pakhtunkhwa, Pakistan. In the present study the genotype data may provide valuable information essential for monitoring the impact of malaria eradication efforts in this region.

## Background

Human malaria is a blood infectious disease caused by mosquito-borne apicomplexan parasites of the genus *Plasmodium* and it is mediated by the arthropod vector *Anopheles* mosquito. This parasite is a unicellular eukaryote that invades host erythrocytes and resides within a parasitophorous vacuole [1]. *P*. *falciparum* is the most virulent of all malaria species associated with high mortality and morbidity rate and exhibits complex genetic polymorphism which may explain its ability to develop multiple drug resistance and avoid vaccines [2].

Pakistan is one of the malaria-endemic countries in Asia for both *P*. *vivax* and *P*. *falciparum* malaria [3, 4]. Pakistan shares its borders with Afghanistan and Iran declared as malaria-endemic countries by WHO, where the cross-border human migrations within these regions increase refugees inflows ultimately causing high malaria transmission in the area [5]. The cross-border movement of people, mutations, and genetic recombination of parasites create new alleles which lead to increased genetic diversity in the parasites population. Reciprocally, human host response to the infection and new drugs choices compromise the frequency of new alleles in the parasite population [6].

Molecular epidemiological studies remain an important tool to analyze the genetic diversity of the *P*. *falciparum* population especially in areas of intense malaria transmission. The strategy to control malarial infection requires an understanding of the genetic composition of *P*. *falciparum*, as this information is essential and may facilitate the development of an effective anti-malarial vaccine [7–9]. In order to determine the number and the types of parasite clones, genotyping is a key approach to determine malarial parasite populations. In molecular epidemiological studies of malaria, this approach is used to analyze the genetic diversity of infections with consideration of different factors like transmission intensity and host immunity. The most widely used technique for malaria genotyping is PCR-based amplification of polymorphic genes encoding the merozoites surface proteins (msp-1 and msp-2) and the glutamate-rich protein (glurp) [10, 11]. The msp-1 and msp-2 are two main *P*. *falciparum* blood-stage malaria vaccine targets [12], which play a key role in the identification of genetically distinct *P*. *falciparum* parasite populations. The msp-1 is a 190 KDa surface protein encoded by *msp-1* gene located on chromosome number 9 and contains 17 blocks of sequences flanked by conserved regions [13, 14]. Whereas its block 2 is a highly polymorphic region, grouped into three sub-allelic families namely K1, MAD20 and RO33 [15]. The msp-2 is also a polymorphic glycoprotein encoded by the *msp-2* gene located on chromosome 2 and comprised of five blocks [16]. The *msp-2* block 3 alleles are grouped into two sub-allelic families FC27 and 3D7 [17]. The glurp being a potential vaccine candidate has been tested in Phase I trial of vaccine development [18] and is expressed in both the erythrocyte stages of the parasite life cycle [19]. Glurp protein-based antibodies can inhibit the growth of *P*. *falciparum* and are suggested to play important role in controlling parasitaemia [20].

To our knowledge, the genetic diversity of *P*. *falciparum* has been extensively studied in different parts of the world but there is very limited information on the genetic diversity of *msp1*, *msp2* and *glurp* genes in our study area. This study is aimed at evaluating the genetic diversity, multiplicity of infection, the level of malaria transmission, and allele frequencies of *msp-1*, *msp-2* and *glurp* in malarial isolates from 9 districts of Khyber Pakhtunkhwa province of Pakistan.

## Methods

### Study area

**Fig 1:**
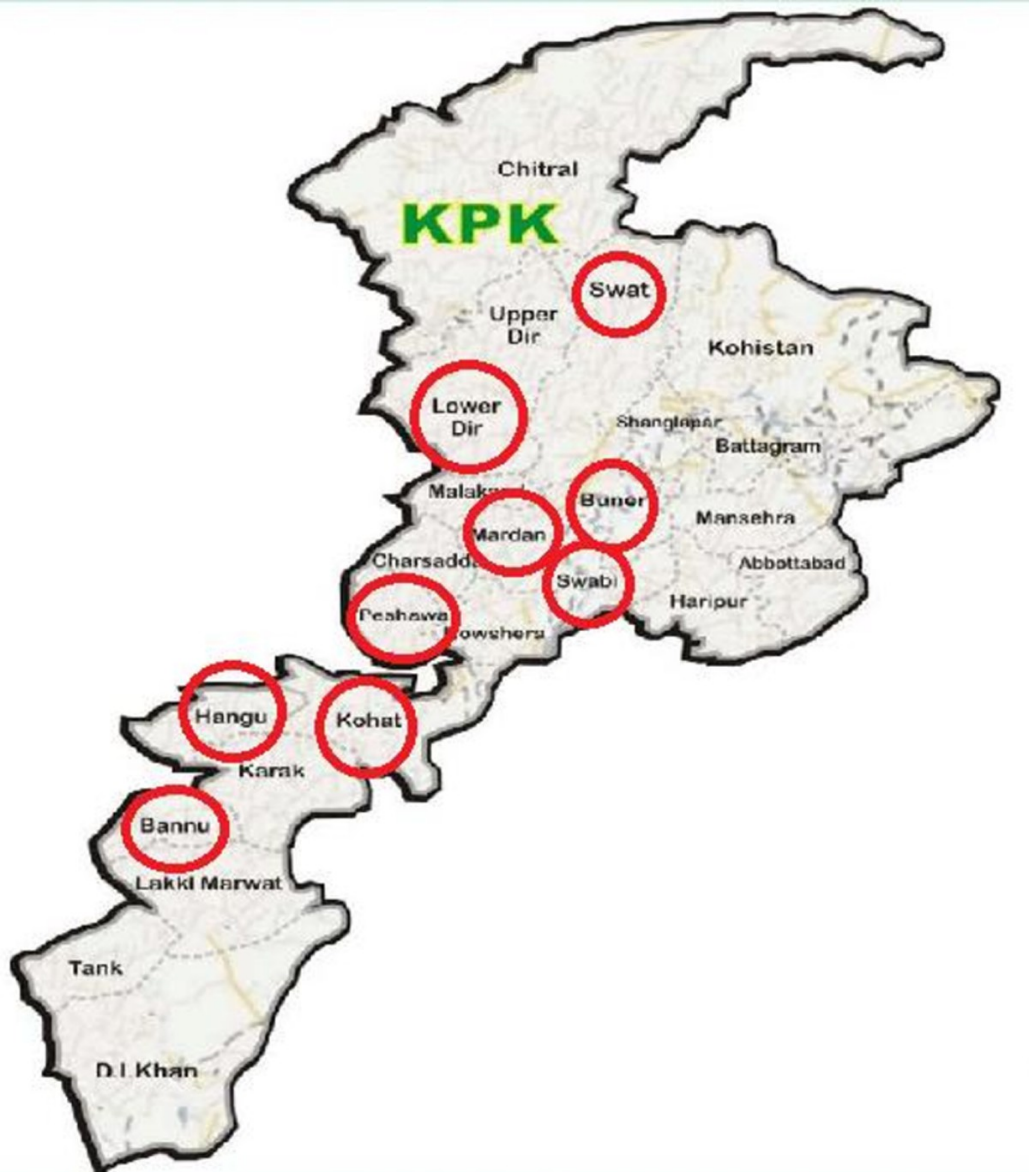
Map of Khyber Pakhtunkhwa showing different districts of study (encircled). Source: Pakistan travel forum; http://www.pakistantravelforum.com/threads/khyber-pakhtunkhwa-kpk.64/.

This study was carried out in 9 districts of Khyber Pakhtunkhwa province including Mardan, Swat, Hangu, Buner, Swabi, Kohat, Bannu, Timergara, and Peshawar. The latitude and altitude of the study site is 34.9526 N and 72.3311 E. The population of the study area is 35.53 million and the total area is 101,741 km^2^. The region receive an annual rainfall of 384 mm from March to May and August to November. The mean temperature ranges from 20°C to 40°C. According to Pakistan Malaria Annual Report 2019 [21], Khyber Pakhtunkhwa Province ranked second in the number of malaria cases (31%) in Pakistan after Sindh province whereas the annual parasite index (API) was 6.3 in the year 2018. Samples for this study were collected during the clinical trial conducted during 2017–2019 in different health facilities of the nine districts (Fig 1).

### Study population and blood samples collection

A total of 100 venous blood samples (3mL) were collected from microscopy-confirmed patients having uncomplicated *P*. *falciparum* malaria after taking written informed consent from the patients or the parents of patients aged below 18 years. Among 100 samples, 85 were confirmed positive for *P*. *falciparum* by species-specific PCR analysis. The patients aged 4 months to 60 years were the residents of the study area who visited the local health centers (hospitals, clinics, laboratories, etc) with body temperature of ≥37.5°C. This study was reviewed and approved by the Ethical Committee of the Department of Biochemistry, Abdul Wali Khan University Mardan (*AWKUM/Biochem/Dept/Commit/18*). The blood was stored at 20°C before DNA extraction at the Biochemistry research lab, Abdul Wali Khan University Mardan, Pakistan. Genomic DNA was extracted from 200µl whole blood samples using the Qiagen DNA extraction kit (Qiagen, Hilden, Germany) following the manufacturer’s instructions.

### Malaria parasite density

Thick and thin blood smears were stained with Giemsa stain for 20 minutes. Parasite density was determined by analyzing the number of asexual parasites per 200 white blood cells and calculated per microliter by multiplying the number of parasites with 8000/200 assuming a blood cell count of 8000 cells per microliter. In case of no result using 200 high power ocular fields of the thick film, it was considered negative [22]. The data were graded into 3 categories according to parasitaemia as patients with 50–5000 parasites/µL, 5000–10000 parasites/µL, and patients with more than 10000 parasites /µL [23].

% parasitaemia = Parasitized RBCs /total number of RBCs X 100

### Multiplicity of infection

The mean multiplicity of infection (MOI) was calculated by dividing the total number of fragments detected in both *msp-1* and *msp-2* loci by the number of samples positive for both markers. The isolates with more than one allelic family were considered polyclonal infections and those with single allelic family were classified as monoclonal infections. The number of infecting genotypes present in each sample represents the multiplicity of infection (MOI) for that sample was calculated as the highest number of alleles obtained in the samples [24, 25].

### Genotyping of *P*. *falciparum msp-1*, *msp-2* and *glurp* genes

Nested PCR genotyping of the polymorphic region of *msp-1* (block-2), *msp-2* (block-3), and *glurp* (RII repeat regions) was performed using primers and methods as previously described [26, 27]. The initial amplification primers pairs corresponding to conserved sequences within polymorphic regions of each gene were used in separate reactions (Table 1). The product generated in the initial amplification was used as a template in subsequent nested PCR. In the nested PCR, primers pairs targeted the respective allelic types of *msp-1* (KI, MAD20 and RO33), *msp-2* (3D7 and FC27) and the RII region of *glurp* with amplification mixture containing 250 nM of each primer (except *glurp* nest 1 primers where 125 nM were used) 2mM of MgCl_2_ and 125 µM of each dNTPs and 01 unit of Taq DNA polymerase. The cyclic condition in the thermocycler (My Cycler- Bio-Rad, Hercules, USA) for initial *msp-1*, *msp-2* and *glurp* PCR were as follows: 5 min at 95°C followed by 30 cycles for 1 min at 94°C, 2 min at 58°C and 2 min at 72°C and final extension of 10 min at 72°C. For *msp-1* and *msp-2*, nested PCR conditions were as follows: 5 min at 95°C, followed by 30 cycles for 1 min at 95°C, 1 min at 61°C and 2 min at 72°C and final extension of 5 min at 72°C [28]. The amplified *msp-1*, *msp-2* and *glurp* fragments were resolved by gel electrophoresis using 2% agarose gel and visualized under UV light using Gel doc system after staining with ethidium bromide. The size of DNA fragments was estimated by visual inspection using a 100 bp DNA ladder marker (New England Biolabs. Inc, UK).

**Table 1:** Basic information of the primers used to amplify *msp-1*, *msp-2* and *glurp* genes of *P*. *falciparum* isolates from Khyber Pakhtunkhwa, Pakistan.

Gene (PCR reaction)	Primers sequence	Annealing Temp	Product size
*msp-1* (N1)	F: 5’-CTAGAAGCTTTAGAAGATGCAGTATTG-3’	55°C	
** **	R: 5’-CTTAAATAGTATTCTAATTCAAGTGGATCA-3’		
**K1 family (N2)**	F: 5’-AAATGAAGAAGAAATTACTACAAAAGGTGC-3’	56°C	100-400bp
** **	R: 5’-GCTTGCATCAGCTGGAGGGCTTGCACCAGA-3’		
**MAD20 family (N2)**	F: 5’-AAATGAAGGAACAAGTGGAACAGCTGTTAC-3’	56°C	100-390bp
** **	R: 5’-ATCTGAAGGATTTGTACGTCTTGAATTACC-3’		
**RO33 family (N2)**	F: 5’-TAAAGGATGGAGCAAATACTCAAGTTGTTG-3’	56°C	100-360bp
** **	R: 5’-CATCTGAAGGATTTGCAGCACCTGGAGATC-3’		
*msp-2* (N1)	F: 5’-ATGAAGGTAATTAAAACATTGTCTATTATA-3’	56°C	
** **	R: 5’-CTTTGTTACCATCGGTACATTCTT-3’		
**FC27 family (N2)**	F: 5’-AATACTAAGAGTGTAGGTGCARATGCTCCA-3’	56°C	150-900bp
** **	R: 5’-TTTTATTTGGTGCATTGCCAGAACTTGAAC-3’		
**3D7/IC family (N2)**	F: 5’-AGAAGTATGGCAGAAAGTAAKCCTYCTACT-3’	56°C	150-900bp
*Glurp*			
**(N1)**	F: 5’-TGAATTTGAAGATGTTCACACTGAAC-3’	55°C	
	F: 5’-GTGGAATTGCTTTTTCTTCAACACTAA-3’		
**(N2)**	F: 5’-TGAATTTGAAGATGTTCACACTGAAC-3’	56°C	600-1100bp
	F: 5’-GTGGAATTGCTTTTTCTTCAACACTAA-3’		

### Statistical analysis

All the statistical analyses were performed using SPSS version 22. The *msp-1*, *msp-2* and *glurp* allelic frequency and their mean MOI were calculated. The spearman’s rank correlation coefficient was calculated to evaluate the relationship of MOI with parasite densities and age groups of the patients. A *P* value of < 0.05 was considered indicative of statistically significant differences.

## Results

### General characteristics of the study population

Out of 100 jhmicroscopy-based confirmed samples of *P*. *falciparum* malaria, 85 PCR-confirmed samples were included in this study. Out of 85 *P*. *falciparum* patients attending health facilities in study area, 12.94% (11/85) were from Swat, 10.6% (09/85) each from Mardan, Peshawar, Timergara and Swabi, 11.8% (10/85) each from Buner, Hangu and Kohat, and 9.42% (08/85) from Bannu. Among the patients, 58.8% (50/85) were male and 41.2% (35/85) female. Subjects of the study aged 4 months to 65 years with a mean age of 33.9±1.6 years. The highest numbers of study subjects [40% (34/85)] were in the age group of 21–40 years while the lowest [1.2% (1/85)] in the age group of >60 years. The parasite density ranged from 3451 to 89,045 parasites/µl with a mean density of 15197 parasites (95% CI (12059–18335) per microliter of blood. The highest mean parasite density was observed in District Swat (22303.45 Parasites/µl) while the lowest in District Bannu (7623.37 Parasites/µl) (Table 2).

**Table 2:** Population characteristics of *P*. *falciparum* infected patients in the study area.

Gender	Mardan n (%)	Swat n (%)	Buner n (%)	Hungo n (%)	Swabi n (%)	Kohat n (%)	Bannu n (%)	Timergara n (%)	Peshawar n (%)	Total
**Male**	7(77.7%)	5(45.5%)	4(40.0%)	6 (60.0%)	4(44.4%)	6(60.0%)	4(50.0%)	7(77.8%)	7(77.8%)	50(58.8%)
**Female**	2(22.3%)	6(54.5%)	6(60.0%)	4 (40.0%)	5(55.6%)	4(40.0%)	4(50.0%)	2 (22.2%)	2(22.2%)	35(41.2%)
**Total**	9(100.0%)	11(100.0%)	10(100.0%)	10(100.0%)	9(100%)	10(100.0%)	8(100.0%)	9(100.0%)	9 (100.0%)	85(100.0%)
**Age group**										
**<5**	1 (11.11%)	0 (0.00%)	1 (10.0%)	1 (10.0%)	0 (0.00%)	1(10.0%)	1 (12.5%)	1 (11.11%)	1 (11.11%)	07 (8.24%)
**5–20**	1 (11.11%)	2 (18.18%)	1 (10.0%)	3 (30.0%)	2 (22.22%)	0 (0.0%)	0 (0.00%)	2 (22.22%)	3 (33.33%)	14 (16.47%)
**21–40**	3 (33.33%)	7 (63.63%)	3 (30.0%)	3 (30.0%)	5 (55.55%)	3(30.0%)	2 (25.0%)	4(44.44%)	4 (44.44%)	34 (40.0%)
**41–60**	4 (44.44%)	2 (18.18%)	5 (50.0%)	3(30.0%)	2 (22.22%)	5 (50.0%)	5 (62.5%)	2 (22.22%)	1 (11.11%)	29 (34.11%)
**>60**	0 (0.00%)	0 (0.00%)	0(0.00%)	0 (0.00%)	0 (0.00%)	1 (10.0%)	0 (00.0%)	0 (0.00%)	0 (0.00%)	01(1.18%)
**Total**	9 (10.59%)	11(12.95%)	10 (11.76%)	10 (11.76%)	9 (10.59%)	10(11.76%)	8 (9.41%)	9 (10.59%)	9 (10.59%)	85 (100.0%)
**Mean Parasitaemia**	20233.83	22303.45	13639.8	9881.1	17358.5	15148.9	7623.37	19119.6	9856.3	

### Frequencies of *msp-1*, *msp-2* and *glurp* allelic families

Out of 85 *P*. *falciparum* positive isolates, successful amplification occurred in 88.23% (75/85) isolates for *msp-1*, 78.9% (67/85) for *msp-2* and 70% (60/85) for *glurp* loci. For *msp-1*, the K1 allelic family was predominant [66.66% (50/75)], followed by RO33 [58.66% (44/75)] and MAD20 allelic family [54.0% (41/75)]. Frequencies of different types of *msp-1* and *msp-2* alleles, their combinations, and multiplicity of infection across the study sites are shown in Table 3. The frequency of samples having only K1, MAD20 and RO33 was 21.3% (16/75), 8% (6/75) and 10.7% (8/75), respectively. The *msp-1* positive samples (40%) were classified as monoclonal infections while the remaining 60% were classified as polyclonal infections with K1/RO33, K1/MAD20 and MAD20/RO33 (dimorphic) representing 13.3%, 12% and 14.7%, respectively. Meanwhile 20% samples exhibited all the three allelic types (trimorphic) together (K1/MAD20/RO33). In *msp-2*, the FC27 and 3D7 allelic families were comparatively close in the frequency of 70.14% (47/67) and 67.16% (45/67), respectively. For *msp-2*, the total monoclonal infections were 62.68% (42/67) in which 29.85% (20/67) samples had only 3D7 allelic family while the samples containing FC27 allelic family were 32.8% (22/67). The samples with polyclonal infection (FC27+3D7) were 37.4% (25/67). For *glurp*, 60/85 (70.6%) samples were found positive for RII repeats region producing 09 distinct sized alleles ranging from 600-1100bp in length among which 700bp allele was predominant [21/60 (35%)] (Table 3).

**Table 3:** Frequencies and MOI of *Plasmodium falciparum msp-1* and *msp-2* isolates.

Allelic Type (*msp-1*)	Mardan n(%)	Swat n(%)	Buner n(%)	Hungo n(%)	Swabi n(%)	Kohat n(%)	Bannu n(%)	Timergara n(%)	Pesh n(%)	Total
**K1**	0	3(30)	3(25)	2(28.6)	0	3(30)	3(50)	1 (16.7)	1(14.3)	16(21.3)
**MAD20**	0	1(10)	2(16.7)	1(14.3)	0	0	0	3(50)	0	06(8.00)
**RO33**	0	0	1(8.3)	2(28.6)	1(12.5)	1(10)	1(16.7)	1(16.7)	1(14.3)	08(10.7)
**K1/MAD20**	2(22.3)	1(10)	2 (16.7)	1(14.3)	0	1 (10)	0	1 (16.7)	0	09(12.0)
**K1/RO33**	3(33.4)	0	1 (8.3)	0	0	1 (10)	1(16.7)	0	4(57.1)	10(13.3)
**MAD20/RO33**	2(22.3)	1(10)	1 (8.3)	1(14.3)	2(25)	2 (20)	1 (16.7)	0	1(14.3)	11(14.7)
**K1/MAD20/RO33**	2(22.3)	4(40)	2(16.7)	0	5(62.5)	2 (20)	0	0	0	15(20.0)
**Total**	9(100)	10(100)	12 (100)	7(100)	8(100)	10(100)	6(100)	6(100)	7(100)	75(100)
**Total K1**	7(77.7)	8(80)	8(66.7)	3(42.8)	5(62.5)	7(70)	4(66.6)	2 (33.3)	5(71.4)	50(66.7)
**Total MAD20**	6(66.6)	7(70)	7(58.3)	3(42.8)	7(87.5)	5(50)	1(16.6)	4 (66.6)	1(14.2)	41(54.7)
**Total RO33**	7(77.7)	5(50)	5(41.6)	3(42.8)	8(100)	6(60)	3(50)	1(16.6)	6(85.7)	44(58.7)
**Multi-clonal isolates**	9(100)	7(70)	6(50.0)	2(28.6)	7(87.5)	6(60)	2(33.3)	1(16.7)	5(71.4)	45(60.0)
**MOI**	**2.2**	**2.0**	**1.8**	**1.0**	**2.2**	**1.8**	**1.0**	**1.0**	**1.3**	**1.8**
Allelic Type (*msp-2*)										
**FC27**	3(42.8)	3(30)	3(42.8)	1(12.5)	1(11.1)	3(30)	0	4(80)	4(57.1)	22(32.8)
**3D7**	3(42.8)	4(40)	2(28.6)	3(37.5)	2(22.2)	2(20)	2(50)	0	2(28.6)	20(29.9)
**FC27/3D7**	1(14.3)	3 (30)	2 (28.6)	4 (50)	6 (66.6)	5(50)	2 (50)	1 (20)	1(14.3)	25(37.3)
**Total**	7(100)	10(100)	7(100)	8 (100)	9(100)	10(100)	4(100)	5(100)	7(100)	67(100)
**Total FC27**	4(57.1)	6(60)	5(71.4)	5(62.5)	7 (77.7)	8(80)	2(50)	5(100)	5(71.4)	47(70.1)
**Total 3D7**	4(57.1)	7(70)	4(57.1)	7 (87.5)	08 (88.8)	07(70)	04(40)	1(20)	3(42.8)	45(67.2)
**Multi-clonal isolates**	1(14.3)	3(30)	2(28.6)	4 (50)	6 (66.6)	5(50)	2(50)	1(20)	1(14.3)	25(37.3)
**MOI**	**1.0**	**1.2**	**1.0**	**1.2**	**1.7**	**1.5**	**1.0**	**1.0**	**1.0**	**1.4**

### Genetic diversity and allelic frequency of *msp-1*, *msp-2* and *glurp* genes

The allelic genotyping data revealed the polymorphic nature of *P*. *falciparum* parasites in Khyber Pakhtunkhwa province. In *msp-1*, *msp-2* and *glurp* gene, different allelic types were identified. Alleles of *msp-1*, *msp-2* and *glurp* were classified according to the size of the amplified PCR bands.

**Fig 2:**
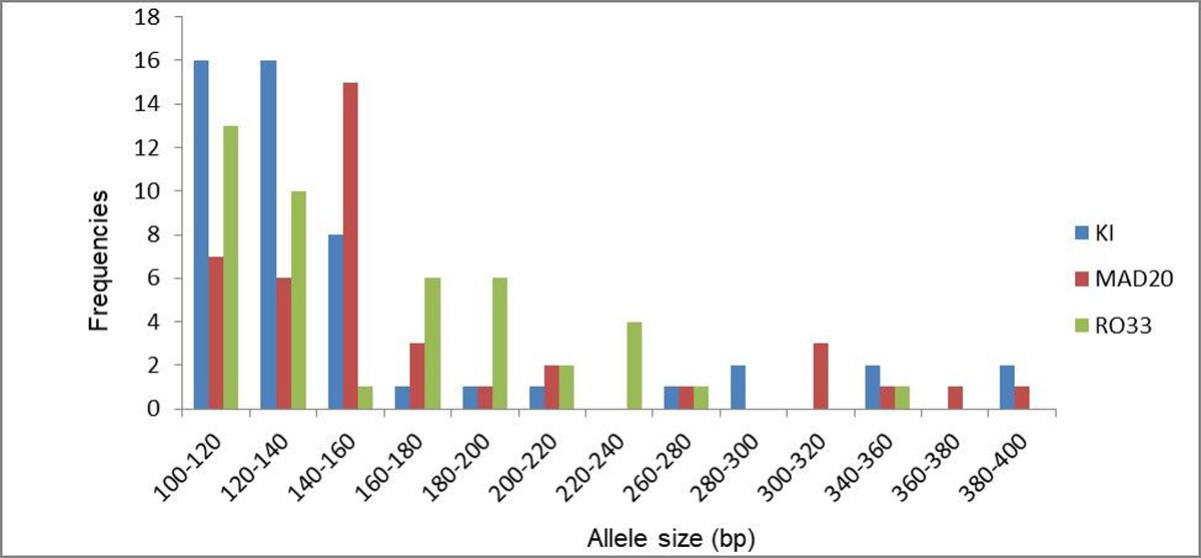
Prevalence of K1, MAD20 and RO33 allelic families of *msp-1* isolates based on alleles sizes (base pairs).

PCR-amplifications were successful in 88.23% (75/85) isolates for *msp-1* gene. Thirty different alleles were detected in *msp-1* gene, among which 10 alleles for K1 (fragments ranged 100-400bp), 11 alleles for MAD20 (fragments ranged 100- 390bp) and 9 alleles for RO33 (fragments ranged 100-360bp). Allelic fragments of 100–140 bp were predominant for both K1 and RO33 allelic families while allelic fragments of 140–160 bp were most frequently prevalent in MAD20 allelic family (Fig 2).

**Fig 3:**
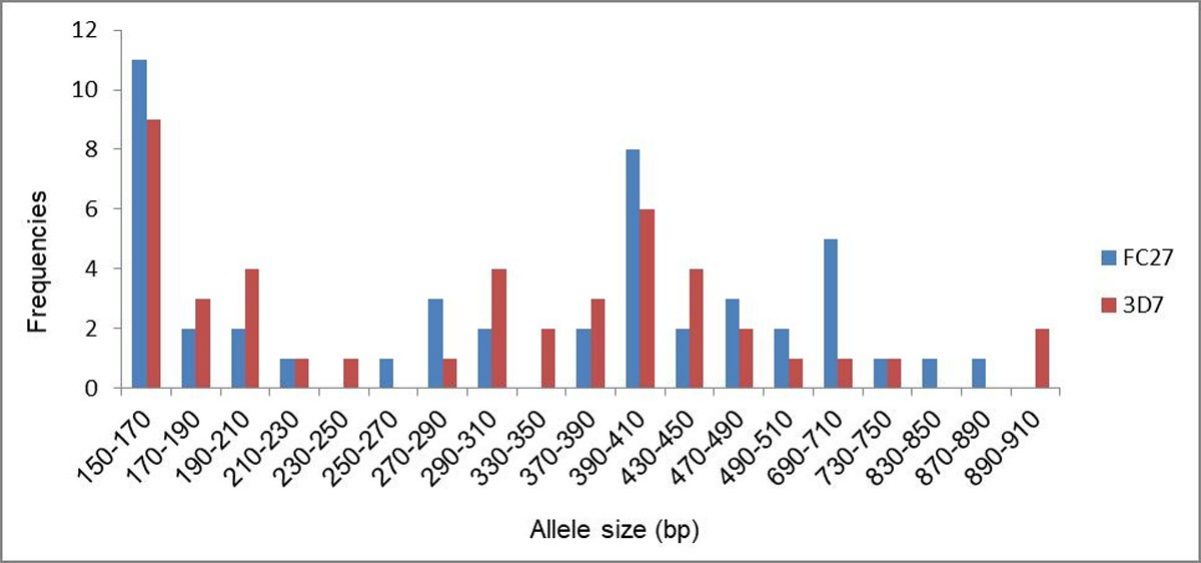
Prevalence of *P*. *falciparum* FC27 and 3D7 *msp2* alleles classified by length (base pair).

Likewise, for *msp-2* gene, 78.9% (67/85) *P*. *falciparum* isolates were successfully amplified and 32 individual alleles were detected including 16 alleles each for FC27 and 3D7 allelic family (Fragments’ size ranged 150-900bp). In these, 150–170 bp and 390–410 bp allelic fragments were predominant for both the *msp-2* allelic families (Fig 3).

**Fig 4:**
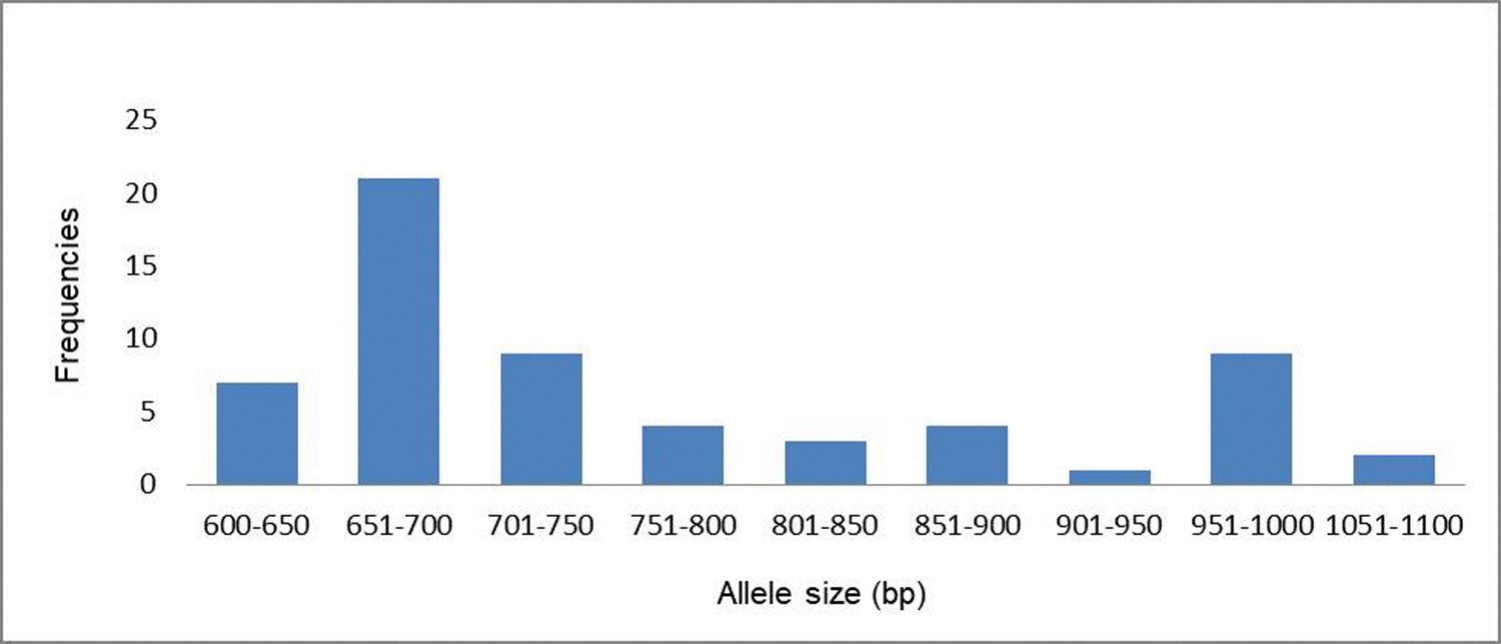
Prevalence of *P*. *falciparum glurp* alleles classified by length (base pairs).

For *glurp* gene in RII repeat region, 60 samples were successfully genotyped resulting in 9 different alleles having fragment sizes ranging from 600 to 1100 bp. Most prevalent allelic fragment was 651bp-700bp (35%) while 901bp-950bp allelic fragment was the least prevalent (1.6%) (Table 4 & Fig 4).

**Table 4:** Distributions of allelic variants of *glurp* RII repeat region of *P*. *falciparum* populations in Khyber Pakhtunkhwa, Pakistan.

Genotype	Allelic variants of *glurp*	N (%)
**1**	600–650	07(11.7)
**2**	651–700	21(35.0)
**3**	701–750	09 (15.0)
**4**	751–800	04 (6.6)
**5**	801–850	03 (5.0)
**6**	851–900	04 (6.6)
**7**	901–950	01(1.6)
**8**	951–1000	09(15.0)
**9**	1051–1100	02 (3.33)

### Multi-clonal infections

The multiplicity of infection (MOI)/number of genotypes per infection was calculated by dividing the total number of fragments detected in one antigenic marker by the number of samples positive for the same marker. Out of 85 samples, 86.1% showed more than one parasite genotype (multi-clonal infections). The overall mean multiplicity of infection for both *msp-1* and *msp-2* genotypes was detected as 1.6. Multiple clones were detected for all the 3 genes under study with 60% (45/75) multi-clonal infections for *msp-1*, 37.3% (25/67) for *msp-2* and 3.34% (2/60) for *glurp* genes. The mean MOI for *msp1*, *msp2* and *glurp* was calculated as 1.8, 1.4 and 1.03, respectively.

### Relationship of MOI with age groups and parasite densities

The prevalence of *msp-1*, *msp-2* and *glurp* allelic families and their sub-allelic types were significantly higher in number among patients’ age group 21–40 years as compared to other age groups. The highest MOI (1.9) for *msp-1* was observed in age groups 21–40 and 41–60 years while the lowest MOI (1.0) was in age group >60 years. Likewise for *msp-2*, the MOI was higher (1.5) in the age group 41–60 years and the lowest (1.0) was detected for the age group >60 years. No statistically significant correlation was found between the multiplicity of infection and age groups of different patients for *msp-1* (Spearman rank coefficient = 0.050; *P* = 0.6), *msp-2*, (Spearman rank coefficient = 0.094; *P* = 0.3) and for *glurp* genes (Spearman rank coefficient = 0.036; *P* = 0.7) (Table 5). However, an increasing trend of mean MOI of individual *msp-1*, *msp-2* and their overall MOI was observed with the increase in the age of patients below 60 years. The patients with age>60 years were very few and therefore all the genes revealed the lowest MOI values for this age group.

**Table 5:** Distribution of *msp-1*, *msp-2* and *glurp* allelic types among different age groups of *P*. *falciparum* infected patients from KP, Pakistan.

Genes	Allelic type	<5	5–20	21–40	41–60	>60	Total
***msp-1***	K1	01(1.3)	0	07 (9.3)	08 (10.7)	0	16 (21.3)
	MAD20	01(1.3)	04 (5.3)	01(1.3)	00	0	06 (8.00)
	RO33	02(2.7)	01 (1.3)	03 (4.0)	01 (1.3)	01 (1.3)	08 (10.7)
	K1/MAD20	0	03 (4.0)	02 (2.7)	04 (5.3)	0	09 (12.0)
	K1/RO33	01(1.3)	01(1.3)	05 (6.7)	03 (4.0)	0	10 (13.3)
	MAD20/ RO33	01(1.3)	01(1.3)	06 (8.0)	03 (4.0)	0	11 (14.7)
	K1/MAD20/RO33	0	02(2.7)	08 (10.7)	05 (6.7)	0	15 (20.0)
	Total	06 (8.0)	12 (16.0)	32 (42.7)	24 (32.0)	01 (1.3)	75 (100)
	Total K1	02 (33.3)	06 (50.0)	22 (68.7)	20 (83.3)	0	50 (66.7)
	Total MAD20	02 ((33.3)	10 (83.3)	17 (53.1)	12 (50.0)	0	41 (54.7)
	Total RO33	04 (66.7)	05 (41.7)	23 (71.9)	12 (50.0)	01	44 (58.7)
	Multi-clonal Isolates	02 (2.7)	07 (9.3)	22 (29.3)	15 (20.0)	0	45 (60.0)
	MOI	**1.4**	**1.7**	**1.9**	**1.9**	**1.0**	**1.8**
***msp-2***							
	FC27	01(1.5)	04 (6.0)	08 (12.0)	08 (12.0)	01 (1.5)	22 (32.8)
	3D7	01 (1.5)	03 (4.5)	11 (16.4)	05 (7.5)	0	20 (29.9)
	FC27/3D7	01 (1.5)	04 (6.0)	09 (13.4)	11 (16.4)	0	25 (37.3)
	Total	03 (4.5)	11 (16.4)	28 (41.8)	24 (35.9)	01(1.5)	67 (100)
	Total FC27	02(66.7)	08 (72.7)	17 (60.7)	19 (79.2)	01 (100)	47 (70.1)
	Total 3D7	02(66.7)	07 (63.6)	20 (71.4)	16 (66.7)	0	45 (67.2)
	Multi-clonal Isolates	01(1.5)	04 (6.0)	09 (13.4)	11 (16.4)	0	25 (37.3)
	MOI	**1.4**	**1.4**	**1.3**	**1.5**	**1.0**	**1.4**
	Overall Mean MOI	**1.4**	**1.6**	**1.6**	**1.7**	**1.0**	**1.6**
***glurp***							
		07	10	21	23	01	62
	MOI	**1.1**	**1.0**	**1.05**	**1.0**	**1.0**	**1.03**

The distribution of *msp-1* gene and its sub-allelic families according to parasitaemia revealed correlation differences to some extent but not significant (Spearman rank 0.205; *P* = 0.060). Likewise, for *glurp* gene and its alleles, this association was non-significant showing the same MOI for all parasitaemia groups (0.026; *P* = 0.814). On the other hand, MOI and parasite density for *msp-2* were significantly correlated (Spearman rank coefficient 0.250; *P* = 0.021). The MOI and parasite density of all the markers collectively (*msp-1*, *msp-2* and *glurp*) was not significantly correlated (*P* = 0.23). For *msp-1*, the highest MOI (2.1) was observed in the range of 50–5000 parasitaemia and the lowest MOI (1.5) in 5001–10000 parasite density. For *msp-2*, lower MOI (1.2) was detected for 50–5000 parasitaemia and higher MOI (1.5) for >10000 parasitaemia. The distribution of different alleles of *msp-1*, *msp-2* and *glurp* according to parasitaemia is shown in Table 6).

**Table 6:** Distributions of *msp-*1 (blocks-2), *msp-2* (block-3) and glurp RII allelic types among parasitaemia groups in the study area.

***msp-1***	50–5000	5001–10000	>10000	Total
K1	0	10(13.3)	6(8.0)	16(21.3)
MAD20	1(1.4)	1(1.4)	4(5.3)	06(8.00)
RO33	0	5(6.7)	3(4.0)	08(10.7)
K1/MAD20	3(4.0)	2(2.6)	4(5.3)	09(12.0)
K1/RO33	1(1.4)	6(8.0)	3(4.0)	10(13.3)
MAD20/ RO33	1(1.4)	2(2.6)	8 (10.7)	11(14.7)
K1/MAD20/RO33	2(2.6)	2(2.6)	11(14.7)	15(20.0)
Total	8(10.8)	28(37.2)	39(52.0)	75(100)
Total K1	6 (75.0)	20(71.4)	24(61.5)	50(66.7)
Total MAD20	7 (87.5)	7(25.0)	27(69.2)	41(54.7)
Total RO33	4 (50.0)	15(53.6)	25(64.1)	44(58.7)
Muticlonal isolates	07(9.3)	12(16.0)	26(34.6)	45(60.0)
MOI	2.1	1.5	1.7	1.8
***msp-2***				
3D7	3(4.5)	8(11.9)	11(16.4)	22 (32.8)
FC27	2 (3.0)	10 (15.0)	08 (11.9)	20 (29.9)
3D7+FC27	2 (2.9)	7(10.4)	16(23.9)	25 (37.3)
Total	7 (8.9)	25(37.3)	35 (53.7)	67(100)
Total 3D7	5 (66.7)	15(60.0)	27(75)	47 (70.1)
Total FC27	4 (50.0)	17(68.0)	24 (69.4)	45 (67.2)
Multi-clonal isolates	02(1.5)	07(10.4)	16(23.9)	25 (37.3)
MOI	1.2	1.3	1.5	1.4
Overall MOI	1.6	1.4	1.6	1.6
***glurp***	9(15)	20(33.4)	31(51.6)	60 (100)
MOI	1.0	1.0	1.0	1.03

## Discussion

The present study provides a detailed analysis and assessment of genetic diversity and polymorphism of *P*. *falciparum* isolates in Khyber Pakhtunkhwa, Pakistan. The purpose of this study was to determine the genetic diversity and molecular characterization of *P*. *falciparum* isolates using their different vaccine candidates genes *viz*. *msp-1*, *msp-2*, *and glurp* from nine districts of Khyber Pakhtunkhwa province. Genetic diversity and polymorphism play an important role in the acquisition of anti-malaria parasite immunity [29, 30], to identify the genotypes circulating in different geographical settings and to effectively monitor malaria control measures.

The allele specific genotyping of *msp-1*, *msp-2* and *glurp* in *P*. *falciparum* isolates revealed high allelic diversity in the nine districts of Khyber Pakhtunkhwa. This trend of high allelic diversity in the study area may be attributed to high-level transmission of malaria and subsequent exposure of inhabitants to mosquito bites. This suggests that the occurrence of mixed infections in a population is affected by the intensity of transmission. Nevertheless, there is a possibility of underestimating the exact number of alleles due to limitations in the analytical techniques used in genotyping as it is difficult to distinguish the fragments with length differences of less than 20bp as distinct alleles [17]. This low to moderate rate of MOI in the present study may be due to intensified malaria control interventions by WHO and low migration inflows across the border from Afghanistan during the last decade due to stability and peace in Afghanistan.

In our study, the genetic diversity of *msp-1* and *msp-2* alleles were higher than *glurp* alleles. This result is in the line with the previous studies [24, 31]. Among the allelic families of *msp-1* gene, a total of 30 allelic types were detected in which K1 was the predominant allelic type. This was in close agreement with those reported for Southwest Ethiopia [17], Cote d’ Ivoire and Gabon [32], Central Africa, Gabon, Benin and Ghana [26, 28, 33] where K1 was the highly prevalent allelic family. In contrast, the isolates from Indonesia [34], Malaysia [35] and Sudan [36] exhibited MAD20 allelic family as the most dominant one. These differences in the prevalence of *msp-1* alleles among different studies likely reflect the variation in geographical locations [32, 37].

For *msp-2*, a total 32 allelic types were detected, among which the alleles belonging to 3D7 family and FC27 were equal in number (16 each). This finding is in close agreement with the studies reported from Kenya [13], Congo Brazzaville [38], Peru [29], Iran [30] and other sub-Saharan African countries [39], while it is in disagreement to the findings from Osogbo Nigeria [40] and North-Eastern Myanmar [41] where FC27 was reported as the more prevalent allelic family of *msp-2* gene. For *glurp* gene, the RII region of the *glurp* also showed a high degree of polymorphism in the parasite population of *P*. *falciparum* across Khyber Pakhtunkhwa with 9 different allelic fragments detected. The high number of *glurp* alleles indicates a high malaria endemicity which is also in agreement with the previous studies [39, 42].

For *msp-1*, *msp-2* and *glurp*, the high level of genetic diversity is compatible with the high level of malaria transmission. A total of 30, 32 and 9 distinct alleles for *msp-1*, *msp-2* and *glurp*, respectively, were obtained in isolates from 9 districts of Khyber Pakhtunkhwa. In this study, the genetic diversity found was higher in contrast to those reported from countries like Nigeria (where only 5 and 15 alleles were identified for *msp-1* and *msp-2* genes, respectively) [8], Brazzaville of the Republic of Congo (where 15 and 20 alleles were detected for *msp-1* and *msp-2*, respectively) [43] and Bangui of Central African Republic (for *msp-1* = 17 and for *msp-2* = 25 alleles) [44]. However, the number of alleles in our study were lower than those in Gabon (*msp-1*: 39; *msp-2*: 27) [45]. For *glurp* gene the same result was reported for Northwest Ethiopia (9 *glurp* alleles) [46] and from India where 8 *glurp* alleles were recorded [47]. Conversely, studies from several regions have reported higher polymorphism frequency in the RII region of *glurp* gene than our reported one, for instance, 11 *glurp* genotypes have been reported from Southwestern Nigeria [48] and 14 genotypes from Sub-Sahara Africa [39].

Most of the infections were multi-clonal (60%) in *msp-1* family while *msp-2* isolates were observed with fewer multi-clonal infections (37%) than monoclonal infections (63%). The overall mean multiplicity of infection was 1.6 for both *msp* genes, while the individual multiplicity of infection was detected as 1.8 for *msp-1*, 1.4 for *msp-2* and 1.03 for *glurp* gene. MOI reported in this study was higher than reported from countries like Malaysia where the MOI of *P*. *falciparum* infection for *msp-1* was 1.37 and for *msp-2* was 1.20 [49]. However, the MOI for *msp-2* was lower than in Cote d’ Ivoire (MOI = 2.8) [50]. The overall MOI in our study was 1.6, which is in close agreement with the studies previously conducted in Southern Ghana (MOI = 1.48) [51], Republic of Congo (MOI = 1.7) [45], Southwest Ethiopia (1.8) [17], while it was lower than Northwest Ethiopia (MOI = 2.6) [46], Republic of Congo (2.2) [38], Guinea (5.51) [52], Southwestern Nigeria (2.8) [47] and Mauritania (3.2) [53]. This inconsistency in the pattern of allele frequencies in these very important vaccine candidate genes (*msp-1*, *msp-2 and glurp*) along with drug resistance genes of malarial parasites may be due to geographical variations, their transmission pattern, use of different malarial drugs, and also samples population determination. When there is a higher malaria transmission level, there will be more chances of getting a higher MOI and mean number of alleles per locus.

It was also found that 81% of samples harbored multi-clonal infections (having more than one allele types) which is in line with the previous studies from Mauritania with 82.3% multiple infections [53], Brazzaville Republic of Congo (83%) [38] and Iran (87%) [30]. Conversely, our reported multiple infections cases were higher than those reported from Nigeria (70%) [47], Southwest Ethiopia (59%) [17] and Sudan (62%) [9] showing a lower frequency of samples with multi-clonal infections. Also observed in this research was a high-level prevalence of multi-clonal infections of *msp-1* and *msp-2* genotypes. This high level of genetic diversity is an indication that the parasite population size remained high enough to allow better mixing of different genotypes and also human migration brought a change in genetic diversity by introducing an additional number of parasites alleles [54]. Age is a key factor affecting MOI in *P*. *falciparum* infection and is also involved in the acquisition of immunity against *P*. *falciparum* species [55]. In young patients, a low level of immunity may be the major factor contributing to their vulnerability to control the infection [41, 56]. The high level of malarial transmission in the region may be expected to lead to a higher risk of severe malaria in younger patients where immunity is lower [57]. In our study, MOI was not correlated with patients’ age and the same association has been reported by studies conducted in other countries like Sudan [11], Ethiopia [33], Benin [58] and Senegal [59] which suggests that the MOI is not directly related to the period of acquisition of immunity in asymptomatic patients but reflects the exposure of subjects to malaria in the endemic area. Conversely, our findings disagree with those results reported from Central Sudan [9], Burkina Faso [60] and Bioko Island, Equatorial Guinea [52] where MOI has been reported to be linked with the age of malarial patients. However, partial positive correlations between MOI and parasite density for one of these genes have been reported by studies from Congo Brazzaville [38, 59] and West Uganda [61].

Among the categorized three groups of parasite density i.e. 50–5000, 5001–10000 and >10000 parasite/µL, we found that there was no significant association between MOI and parasitaemia (*P* = 0.23). This trend is in congruity with previous studies reported from Benin [58] and Nigeria [40]. In contrast to our findings, different studies have shown a direct relationship between the MOI and parasite density like studies conducted in Congo, Brazzaville [38] and West Uganda [61] where the MOI showed an increase accordingly to the increase in parasite densities. These results are consistent with many reports demonstrating that high parasite densities increase the probability of detecting concurrent clones in individuals [61]. This finding reveals that high malaria transmission and also parasite density may have a strong association with the genetic diversity of *P*. *falciparum* in the Khyber Pakhtunkhwa province of Pakistan.

## Conclusions

Field isolates of *P*. *falciparum* in nine districts of Khyber Pakhtunkhwa are highly diverse based on allelic variation in *msp-1*, *msp-2* and *glurp* genes. This study provides basic information on the genetic diversity and multiple infections of *P*. *falciparum msp-1*, *msp-2* and *glurp* isolates important for future studies on dynamics of parasite transmission and to evaluate the malaria control interventions in our study area.
